# Multifocal Cutaneous Angiosarcoma of the Lower Limbs Presenting With Severe Tumour-Related Haemorrhage: A Case Report

**DOI:** 10.7759/cureus.119124

**Published:** 2026-11-04

**Authors:** Shahzad Ahmad, Sonya Morara, Tshegofatso Raborokgwe, Hussain Samuaan, Victoria Weir

**Affiliations:** 1 Respiratory Medicine, Aberdeen Royal Infirmary, Aberdeen, GBR; 2 Plastic Surgery, Aberdeen Royal Infirmary, Aberdeen, GBR; 3 Geriatrics, Aberdeen Royal Infirmary, Aberdeen, GBR; 4 Diabetes and Endocrinology, Aberdeen Royal Infirmary, Aberdeen, GBR; 5 General Medicine, Aberdeen Royal Infirmary, Aberdeen, GBR

**Keywords:** angiosarcoma, cutaneous malignancy, palliative radiotherapy, pulmonary embolism (pe), transfusion-dependent anaemia, tumour bleeding

## Abstract

Cutaneous angiosarcoma (CAS) is a rare and highly aggressive vascular tumour of endothelial origin with a poor prognosis. It can present with atypical skin lesions, making early diagnosis challenging. We present a case of a man in his late 80s who presented with a two-year history of weight loss, fatigue, iron-deficiency anaemia, and persistently elevated inflammatory markers. He subsequently developed rapidly progressive, multifocal violaceous nodules affecting both lower limbs. Initial differential diagnoses included melanocytic and nodular vasculitis. Skin biopsy confirmed high-grade CAS. The lesions progressed to fungation with recurrent bleeding, resulting in severe symptomatic anaemia requiring repeated blood transfusions. Management was complicated by his background of a persistent endoleak after endovascular aneurysm repair (EVAR) of a large abdominal aortic aneurysm (AAA). This case highlights the importance of considering CAS in rapidly evolving, atypically located skin lesions. Early diagnosis and patient-centred multidisciplinary management are essential, particularly when advanced disease and significant comorbidities limit therapeutic options.

## Introduction

Angiosarcomas are a rare and aggressive subtype of soft tissue sarcomas that arise from endothelial cells lining blood and lymphatic vessels [1]. They represent about 2% of all soft tissue sarcomas, with the most common type being cutaneous angiosarcomas (CAS) [2]. Although they can arise in any anatomical location, most CAS lesions arise in the head and neck and typically present as enlarging violaceous patches or nodules [1,3]. Early stages of disease may mimic benign conditions such as bruising or haemangioma, potentially resulting in delayed diagnosis and treatment [1,3]. 

We present a rare case of advanced multifocal CAS in an elderly man with rapidly progressive multifocal lower limb lesions and recurrent tumour-related bleeding.

## Case presentation

A man in his late 80s presented with a two-year history of unintentional weight loss and fatigue. His past medical history included type 2 diabetes mellitus, hypertension, and a large abdominal aortic aneurysm (AAA) previously treated with endovascular aneurysm repair (EVAR) and complicated by persistent endoleak. Bloods showed iron-deficiency anaemia and persistently elevated inflammatory markers. There was no evidence of infection, and inflammatory markers remained elevated despite courses of antibiotics.

The patient did not complain of melena; digital rectal examination was normal, and Quantitative Faecal Immunochemical Test (qFIT) was negative. Computed tomography (CT) of the thorax, abdomen and pelvis did not identify an underlying cause for his symptoms.

During this period, the patient developed purplish nodules on both lower limbs (Figure 1A). These lesions were non-tender on palpation but caused discomfort when sitting down. Over a few months, the nodules increased rapidly in both number and size (Figure 1B).

**Figure 1:**
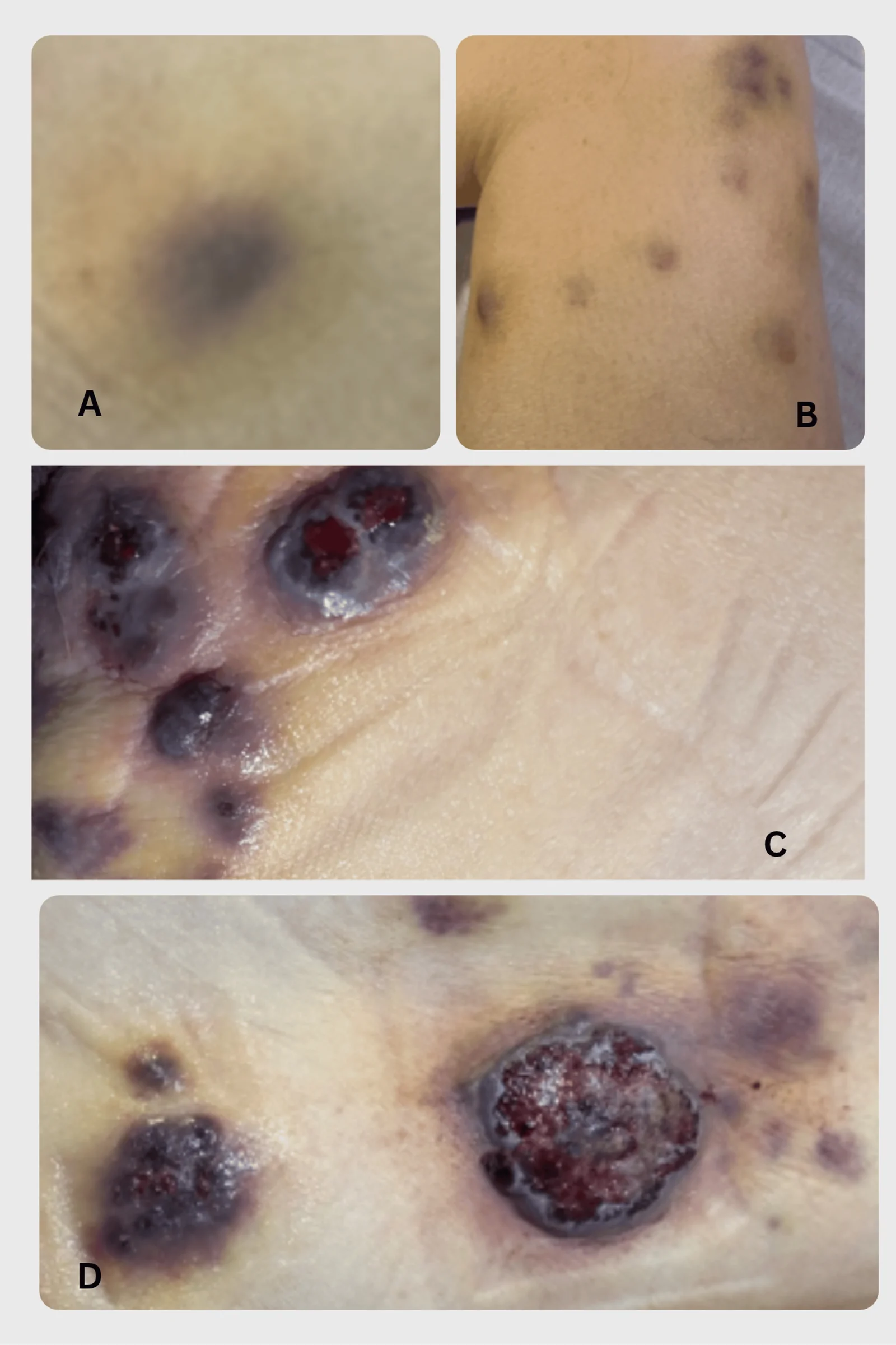
Clinical progression of multifocal cutaneous angiosarcoma. A: initial violaceous nodule; B: progressive development of multifocal violaceous lesions over subsequent months; C, D: fungating lesions with associated bleeding.

The patient was discussed with the dermatology team, where the differentials included melanocytic lesions or inflammatory conditions such as nodular vasculitis. A biopsy was performed of the anterior thigh, confirming high-grade angiosarcoma.

This histology section shows skin containing a dermal-based infiltrative tumour composed of atypical epithelioid cells dissecting the collagen bundles, associated with prominent haemorrhage and brown pigment deposition. Mitotic figures are easily identified. Vague areas suggestive of angiogenesis were present, although well-formed vessels were not identified. Immunohistochemistry demonstrated diffuse, strong staining for ERG within the tumour cells, with patchy staining for CKAE1/3. The tumour cells were negative for Melan-A, S100, SOX10, HMB45 and PRAME. Overall, these features were consistent with Grade 3 angiosarcoma.

The patient was discussed at the National Sarcoma MDT. Given the pathology of high-grade angiosarcoma, the presence of a very large inoperable AAA, and advanced multifocal disease with multiple new lesions appearing rapidly, further active oncological intervention was not considered appropriate. A decision was therefore made for supportive treatment, with a focus on symptom control and optimisation of quality of life.

The patient was prescribed morphine to help manage pain from the lesions, particularly when they were knocked or subjected to contact. Advice was provided regarding the protection of the lesions, and the use of anti-inflammatory gel was also suggested. No further imaging was carried out, as it was unlikely to alter the management.

Clinical progression

The lesions progressed with the development of fungation and recurrent bleeding (Figures 1C, 1D). He developed bleeding from lesions on the left thigh, resulting in worsening symptomatic anaemia requiring multiple blood transfusions. The patient's haemoglobin levels declined over the course of the disease, with marked fluctuations during the later stages (Figure 2).

**Figure 2:**
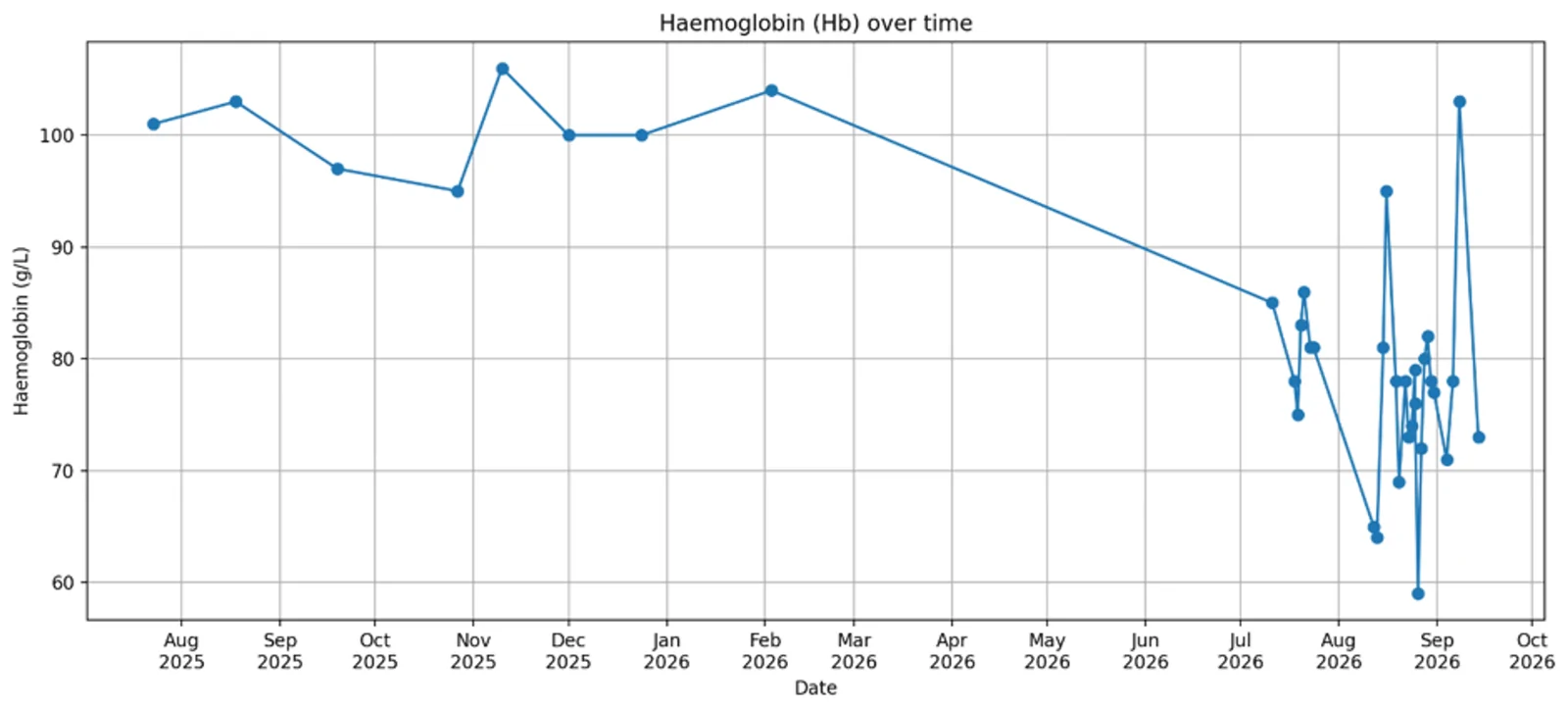
Haemoglobin trend demonstrating progressive anaemia and marked fluctuations during the clinical course.

It was noted that the lesions progressed, resulting in fungation and bleeding. The patient received a single fraction of palliative radiotherapy to an area of angiosarcoma on the posterior thigh with the primary aim of controlling profuse bleeding from the lesions. During hospital admission, it was noted that there was ongoing bleeding from areas of the right thigh, which was managed with dressings. It was felt that further radiotherapy should only be considered if the lesion is causing significant bleeding or pain. As the radiotherapy had not stopped the bleeding from the posterior thigh, it is unlikely to be helpful for other lesions.

During his most recent admission, he developed haemoptysis and a new oxygen requirement. His Wells score was 6.5 [4]. A CT pulmonary angiography (CTPA) was done to see if new symptoms were due to PE or pulmonary spread of angiosarcoma. CTPA showed bilateral lower-lobe segmental pulmonary emboli (Figure 3). It also showed moderate bilateral pleural effusions with associated atelectatic and consolidative changes.

**Figure 3:**
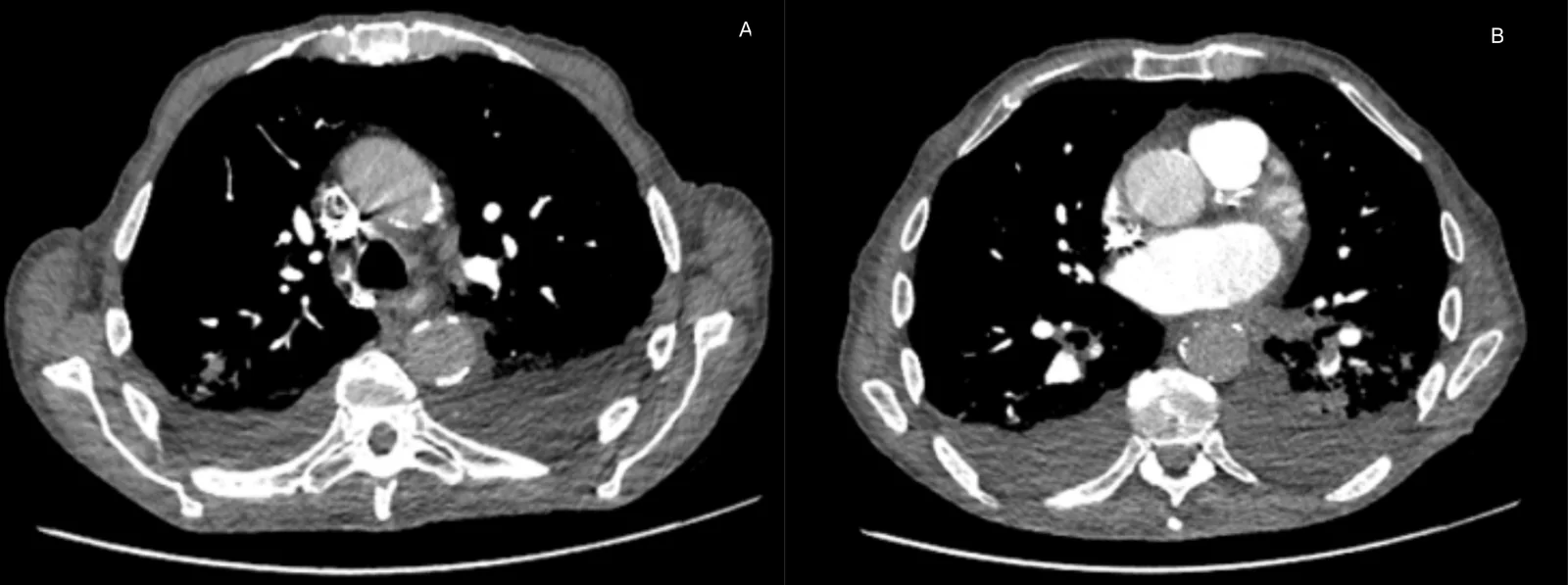
CT pulmonary angiography (CTPA) findings. A: demonstrates right-sided lower-lobe segmental pulmonary emboli; B: demonstrates left-sided lower-lobe segmental pulmonary emboli.

Given anaemia and shortness of breath secondary to ongoing bleeding from the right thigh angiosarcoma, CT angiography of the lower limbs was carried out (Figure 4). This was performed to assess whether the patient was suitable for intervention. It showed progressive disease with new subcutaneous and cutaneous lesions. There were no identifiable feeding vessels or target for embolisation in relation to the lesion in the posterior right thigh.

**Figure 4:**
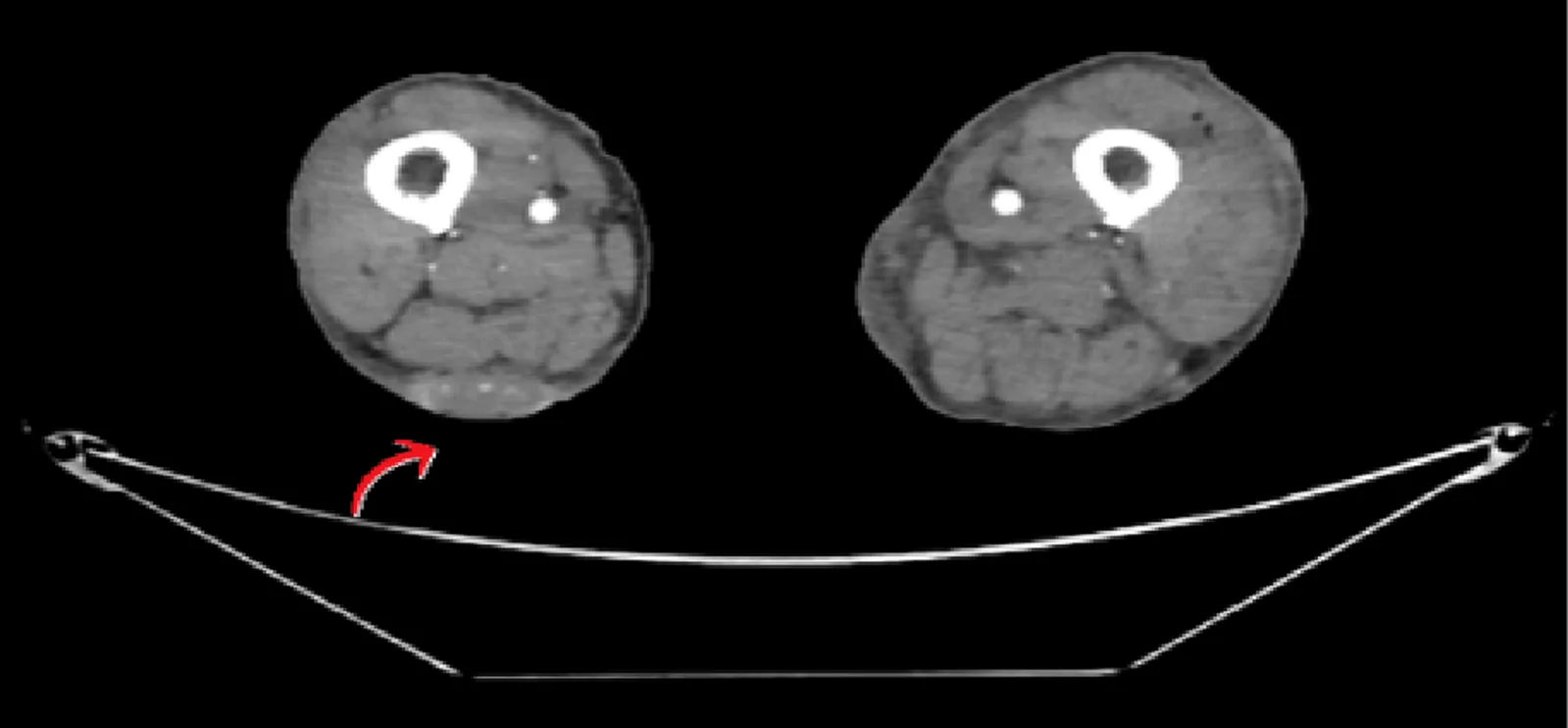
CT angiography of the lower limbs. The arrow is pointing to a cutaneous angiosarcoma lesion.

Management therefore remained supportive, with blood transfusions for symptomatic relief. The patient was transferred to a specialist palliative care unit where he continues his treatment.

## Discussion

CAS is a rare and aggressive malignancy of vascular and lymphatic endothelial cells that accounts for approximately 2% of soft tissue sarcomas [1]. As a broader disease entity, angiosarcoma can arise in multiple tissue sites around the body, while CAS demonstrates a pattern commonly affecting the head and neck region, particularly the scalp [1,2]. It predominantly occurs in elderly male patients, with a noted poor prognosis secondary to its aggressive nature and often late diagnosis.

CAS may occur as a primary malignancy or a secondary malignancy. Primary CAS tends to affect the head and neck, although it can still occur at body extremities. Secondary CAS is associated with recognised risk factors, most commonly secondary to radiation exposure in patients with previous cancer treatment, or with chronic lymphoedema. CAS associated with chronic lymphoedema is known as Stewart-Treves syndrome and was described in patients who developed upper limb lymphoedema following surgical excision of breast cancer and axillary node dissection [3].

CAS has a highly variable presentation, with its early-stage presentation ranging from lesions that mimic a bruise to purple-red nodules or bleeding ulcers [1]. Delays in diagnosis can occur due to features that can be mistaken for benign lesions such as rosacea or eczema. With disease progression, lesions can become multifocal, ulcerated with subsequent haemorrhage. Our patient demonstrated an atypical distribution, presenting with purple-coloured nodules on his lower limbs, which were painful when in contact with other objects. He developed severe anaemia as a result of these lesions bleeding. When discussed with dermatology, initial differentials included melanocytic lesions or inflammatory conditions such as nodular vasculitis.

Several imaging modalities, such as magnetic resonance imaging and computed tomography scans, are often used in the diagnostic workup of CAS and also play an important role in disease extent evaluation.

Definitive diagnosis is established by skin biopsy with histopathological assessment, supported by immunohistochemistry [1]. Histopathology of CAS can be challenging to differentiate from benign vascular tumours, particularly in cases of well-differentiated CAS lesions. They both demonstrate extensive vascular channels lined by endothelial cells; however, features favouring diagnosis of CAS include vascular sinusoids that dissect through collagen bundles, or epithelioid cells that have no definitive vasoformation [5]. Immunohistochemistry supports the diagnosis of CAS, with endothelial markers such as CD31 and ERG commonly used to confirm vascular differentiation [6].

Treatment options for CAS depend on disease extent, requiring a multidisciplinary team to decide on the most suitable options per case. A crucial treatment option for patients with localised disease is wide surgical excision with negative margins [1]. Tumour sizes> 5 cm, positive surgical resection margins, and a depth of invasion> 3 mm are features associated with poorer outcomes and prognosis and an increased rate of disease recurrence once surgically excised [7]. However, given the highly infiltrative nature of CAS, achieving adequate surgical margins can be challenging.

High recurrence rates are also noted in cases where surgical therapy was the only treatment offered. Surgery is therefore not recommended as a stand-alone therapy but is usually used in conjunction with radiotherapy [1]. Radiotherapy was initially utilised in palliative management of CAS, but now plays a role in adjuvant therapy and curative treatment in patients who are not appropriate surgical candidates.

The goal of palliative radiotherapy is to reduce tumour burden and improve symptom control, including pain and uncontrolled bleeding. Our patient was offered palliative radiotherapy to help control bleeding from the lesions. A retrospective study of 31 patients with scalp CAS who underwent treatment with radiotherapy was conducted; of this cohort, five patients presented with bleeding from the cutaneous lesions, with haemostasis achieved in four of these five following radiotherapy [8]. 

Patients with metastatic, recurrent or unresectable CAS can be offered systemic therapies. There are several systemic drug therapies available for the management of CAS, with one particular notable agent being paclitaxel, a taxane-based chemotherapy. It works by ceasing cancer cell proliferation by inhibiting cell division of tumour cells, and it displays antiangiogenic effects which limit blood supply to tumour cells. Its efficacy was demonstrated in the ANGIOTAX phase II trial, which reported progression-free survival rates of 74% at a two-month time point and 45% at a four-month time point [9].

Personalising treatment options offered to patients with CAS is crucial as their demographic, functional status and comorbidities affect which therapies will be appropriate for them. Our patient’s background included a large AAA with a previous EVAR, with follow-up imaging revealing a leaking repair. Due to this, a palliative approach was adopted, as he was deemed not fit for further repair. No curative management options were available for our patient, and instead, palliative radiotherapy was offered to aid with pain and help control bleeding.

The lungs are an important site of metastasis in CAS. Pulmonary metastases are among the most common metastatic manifestations of CAS [8] and occur as a result of haematogenous spread leading to the involvement and destruction of pulmonary vasculature [10]. Several malignancies with pulmonary metastases lead to solid pulmonary nodules, whereas CAS pulmonary metastases may present as thin-walled, haemorrhagic or cystic lesions. Haemoptysis is a common presenting feature of this, which should warrant further investigation for potential spread in known cases of CAS. This was relevant in our patient, who developed haemoptysis, although subsequent CTPA revealed a pulmonary embolism rather than lung lesions.

## Conclusions

This case highlights an unusual presentation of CAS with rapidly progressive multifocal lower-limb lesions and significant tumour-related haemorrhage. Early biopsy of rapidly evolving or atypical cutaneous lesions is important to establish the diagnosis. In advanced disease, recurrent bleeding can be difficult to control and may require an individualised multidisciplinary approach focused on symptom management.
